# A finger photoplethysmography waveform during the valsalva maneuver detects changes in left heart filling pressure after hemodialysis

**DOI:** 10.1186/s12882-015-0135-0

**Published:** 2015-08-14

**Authors:** Panagis Galiatsatos, Kapil Parakh, Jennifer Monti, Sumeska Thavarajah, Harriet Aneke-Ogbu, Amaris Watson, Daniel Kim, Nae-Yuh Wang, Tariq Shafi, Harry A. Silber

**Affiliations:** Division of Cardiology, Johns Hopkins University School of Medicine, Johns Hopkins Bayview Medical Center, 4940 Eastern Avenue, Baltimore, MD 21224 USA; Division of Nephrology, Johns Hopkins University School of Medicine, Johns Hopkins Bayview Medical Center, 4940 Eastern Avenue, Baltimore, MD 21224 USA; Department of Medicine, Johns Hopkins University School of Medicine, Johns Hopkins Bayview Medical Center, 4940 Eastern Avenue, Baltimore, MD 21224 USA

**Keywords:** Valsalva, Left ventricular filling pressure, Volume status, Hemodialysis

## Abstract

**Background:**

A noninvasive system for determining left ventricular (LV) filling pressure may help to improve personalized fluid removal goals in hemodialysis patients. We previously showed that the change in photoplethysmography (PPG) pulse amplitude measured by finger PPG during a Valsalva maneuver correlates with invasively measured left ventricular end-diastolic pressure (LVEDP). This key PPG change, the ratio of finger PPG pulse amplitude at end-Valsalva to baseline, is known as the Pulse Amplitude Ratio, PAR. The objective of this study was to determine how PAR changes after fluid removal in hemodialysis.

**Methods:**

We tested subjects with end-stage renal disease, before and after hemodialysis. Each subject performed a Valsalva maneuver with an effort of 20 mmHg for 10 s, guided by the device display. Finger PPG was recorded continuously before and during the maneuver. PAR was calculated automatically.

**Results:**

Twenty-seven subjects (21 Males) ages 25–75 years were tested. Access sites were AV-fistulas of the arm predominantly. Weight decreased from 99.7 ± 36.9 kg to 97.0 ± 36.0 kg (*p* < 0.0003) with an average fluid removal of 3.07 ± 1.08 l. Correspondingly, PAR decreased from 0.74 ± 0.24 to 0.62 ± 0.23 (*p* = 0.003). The change in PAR was correlated with baseline PAR (*r* = 0.48, *p* = 0.01).

**Conclusion:**

An index of left heart filling pressure obtained noninvasively using finger photoplethysmography during the Valsalva maneuver is sensitive enough to detect reductions in filling pressure after fluid removal with hemodialysis. Further studies are warranted to determine if this method can be used to guide fluid removal during hemodialysis.

## Background

Hemodialysis patients undergo volume shifts during their dialysis in an attempt to reach optimal dry weight. The significance of dry weight achievement lies in that fluid accumulation between dialysis has been demonstrated to be a predictor of death and cardiovascular complications [1]. Aiming to achieve ideal dry weight is hindered by the need to tailor volume subtraction to the hemodynamic tolerance of a patient [2, 3]. Patients with left ventricular dysfunction may be even more susceptible to poor outcomes if hemodialysis fails to remove an adequate fluid amount [4]. Further, intradialytic hypotension continues to be a frequent complication in hemodialysis patients, and is also associated with increased morbidity and mortality [5, 6]. Many tools have been investigated for assessing volume status in hemodialysis patients, including standard echocardiography for Doppler indices of that are related to filling pressure [7], hand-carried ultrasound assessment of IVC [8], chest ultrasound to assess lung water [9], serum proBNP [7], central venous oxygenation [10], and bioimpedance [11]. However, no one test has a combination of being noninvasive, convenient, inexpensive, point-of-care, and immediate, and sensitive and specific enough to determine optimal individual volume removal. Left ventricular filling pressure is a direct hemodynamic determinant of left ventricular strain and of systemic hypotension. Therefore, a noninvasive system for determining left ventricular filling pressure could help to improve personalized fluid removal goals in hemodialysis patients.

Assessing cardiac filling pressure clinically is challenging, especially with current non-invasive strategies (e.g. physical exam, blood markers), since many tests are neither sensitive nor specific [12]. The Valsalva maneuver, defined as a sustained straining against a closed upper airway, has shown utility in assessing volume status and cardiac hemodynamic properties [13–16]. Yet, the Valsalva maneuver remains underutilized clinically, likely due to the inability to standardize the expiratory effort and the difficulties obtaining a continuous noninvasive surrogate of a pressure waveform conveniently [16]. Finger photoplethysmography conveniently provides a continuous pressure surrogate waveform during the Valsalva maneuver. We have shown that the pulse amplitude response of a finger photoplethysmography waveform correlates well with invasively measured left ventricular end-diastolic pressure (LVEDP) [17].

Recently, we have developed a hand-held, battery-powered device that guides patients in performing a Valsalva maneuver at 20 mmHg expiratory effort for 10 s while recording finger photoplethysmography. It automatically calculates pulse amplitude ratio (PAR), the amplitude at end-Valsalva divided by the amplitude at baseline. The objective of this study was to determine if PAR is sensitive enough to detect changes in left heart filling pressure that accompany fluid removal in hemodialysis.

## Methods

Participants were recruited from Johns Hopkins Bayview Medical Center and DaVita J.B. Zachary Dialysis Center from February 2012 to July 2013. Eligible patients included those between the age of 20–85 years who are receiving chronic, intermittent hemodialysis as an inpatient at Johns Hopkins Bayview Medical Center or outpatient at DaVita J.B. Zachary Dialysis. Patients were excluded if they had acute onset of renal failure with less than three dialysis sessions during their inpatient stay, if an inpatient required dialysis at the bedside, patients in the intensive care unit, and patients unable or unwilling to perform a Valsalva maneuver. All participants provided written informed consent. The procedures followed were in accordance with the ethical standards of the Helsinki Declaration of 1975, as revised in 2000, and the Johns Hopkins University Institutional Review Board reviewed and approved the protocol.

Participants were tested while in a seated position (outpatient) or in a semi-recumbent position (inpatient), within 15 min prior to their hemodialysis session and again 15 min after their hemodialysis session was completed. The device’s photoplethysmography transducer was attached to either their middle or ring finger on the hand opposite of the hemodialysis access site. For example, if the subject had an arterio-venous (AV) fistula in their right upper extremity, the photoplethysmography transducer was used on the left middle or ring finger (belonging to the extremity without the AV fistula); if the patient had a left subclavian catheter, the right hand was used. A mouthpiece attached to a pressure transducer input into the device was given to the participant to hold in the contralateral hand. Participants were coached using a graphical user interface on the screen of the device. The screen displayed the expiratory pressure signal, and provided upper and lower limits for the participant to maintain an exhalation effort of 20 mmHg for 10 s. The pressure and photoplethysmography signals were recorded simultaneously. A minimum of three and a maximum of six successful expiratory efforts were acquired.

The patient had the same coach before and after hemodialysis. All coaches were trained and had demonstrated the ability to adequately teach participants prior to enrolling formal subjects. Further, the device’s design allows for standardization of the Valsalva performance; therefore, minimizing inter-observer error.

“Years of hemodialysis” was defined as years requiring hemodialysis without discontinuation. For example, if a subject was on hemodialysis in the past and had stopped (e.g. due to a renal transplant or a switch to peritoneal dialysis), then restarted, the time of restarting to the time of recruitment was defined as “years of hemodialysis”. Only five subjects had prior hemodialysis that was discontinued (either due to a kidney transplant or prior peritoneal).

Transduced signals were input into the device. Filtering parameters of the photoplethysmography signal were 10-Hz low pass and 0.01-Hz high pass. An exhalation effort achieved within 3 s and sustained between 18 and 25 mmHg for 10 s was required in order for the device to record the data. If three successful attempts could not be achieved, whether before or after hemodialysis, the subject was documented as “unable to complete”; any data obtained from such a subject was not used towards analysis. Waveforms were analyzed using software accompanying the digital acquisition system. For each Valsalva effort, the amplitudes of three typical cycles of the baseline photoplethysmography waveform were averaged. The amplitude of the cycle just before 10 s into the Valsalva maneuver was measured. We did not use multiple cycles when calculating pulse amplitude during the Valsalva maneuver because the amplitude changes continuously during the maneuver. The average PAR over all the acceptable efforts was obtained before hemodialysis and again after hemodialysis.

All values are reported as mean ± standard deviation unless otherwise specified. We examined the distribution of variables and change in variables by assessing symmetry of box plots. We did not opt to formally test the normality of these variables because of the expected low statistical power of such a test with the sample size we have. We would not have been able to determine whether a non-significant p-value really meant normality or was due to larger than desired type two error. Coefficient of variation was calculated for each set of baseline PAR measurements as standard deviation / mean. The average coefficient of variation was calculated over all the subjects tested. Statistical comparisons between pre-dialysis and post-dialysis variables were performed using paired *t* tests. Relationships between PAR and other variables were ascertained using simple linear regression. Analyses were conducted with SigmaPlot 11.0 (San Jose, CA).

## Results

A total of 29 subjects were recruited between the two sites; however, 2 of the subjects could not complete the required number of successful Valsalva maneuvers and were excluded from the final analysis. Therefore, 27 subjects, of which 6 were female, were reviewed and their data is presented below. The mean age was 53.2 ± 13.4 years, with the range being between 25 to 75 years. Table 1 lists the characteristics of the participants, along with the absolute number of and the percent of participants with that characteristic. The most common comorbidity among the subjects was hypertension (78 %) and all hypertensive subjects were on antihypertensive medications.

**Table 1:** Subject characteristics

Number of subjects	27
Age (yr, ± S.D.)	53.2 ± 13.4
Gender (male)	21
Comorbidities	
Hypertension (%)	21 (78)
Diabetes type 2 (%)	11 (41)
Congestive Heart Failure (%)	6 (22)
Chronic obstructive pulmonary disease (%)	4 (15)
Peripheral Arterial Disease (%)	0 (0)
Coronary Artery Disease (%)	14 (52)
Cirrhosis (%)	1 (4)
Medications	
Calcium Channel Blockers (%)	16 (59)
β-Blocker (%)	20 (74)
Angiotensin-converting enzyme inhibitor (%)	9 (33)
Angiotensin receptor blocker (%)	2 (7)
Nitrate (%)	2 (7)
Thiazide Diuretic (%)	1 (4)
Loop Diuretic (%)	6 (22)
Clonidine (%)	2 (7)
Hydralazine (%)	4 (15)
Active smokers (%)	3 (11)
Years of hemodialysis (median)	1.5
Access site for hemodialysis	
Catheter (Subclavian) (%)	5 (19)
Catheter (Internal Jugular) (%)	1 (4)
Arterio-venous fistula	
Right Upper Extremity (%)	10 (37)
Left Upper Extremity (%)	11 (41)
Able to make any urine (%)	21 (78)

*S.D.* standard deviation, Co-Morbidities, Medications, Smokers, and Access site for hemodialysis are presented as absolute number of subjects followed by % of subjects in parentheses

With regard to the renal characteristics of the subjects (Table 1), the median duration of ESRD treated with hemodialysis was 1.5 years (range 6 months to 5.25 years). The majority of the subjects (78 %) said they could still make urine. The mean ultrafiltration rate was 1.60 ± 0.29 L/h. As for access sites, 21 subjects had arterio-venous fistulas that were being used. Only one subject did not finish the desired preset hemodialysis time, finishing 30 min early due to cramps; however, the subject was still able to perform an expiratory effort within 15 min of discontinuing hemodialysis.

**Fig. 1:**
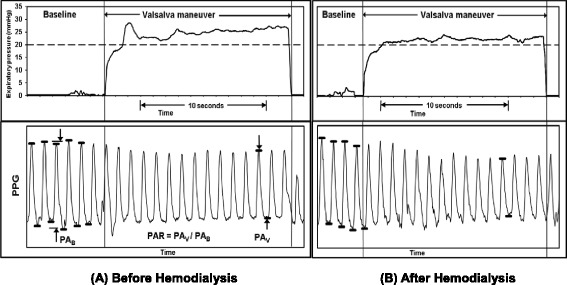
A typical photoplethysmography (PPG) waveform response during the Valsava maneuver: **a** Before hemodialysis and **b** After hemodialysis. Pulse amplitude ratio (PAR) is calculated as the pulse amplitude of the waveform at the end of 10 s of Valsalva (PA_V_) divided by the average pulse amplitude of several cycles at baseline (PA_B_). In this example, PAR = 0.81 before hemodialysis and PAR = 0.67 after hemodialysis. The automated algorithm acquiring the data allows a maximum of 3 s for the subject’s expiratory effort to equal or exceed 20 mmHg in order for the data to be accepted and stored

Figure 1 shows an example of simultaneously recorded photoplethysmography and expiratory pressure effort waveforms. Also shown is how PAR is obtained.

**Fig. 2:**
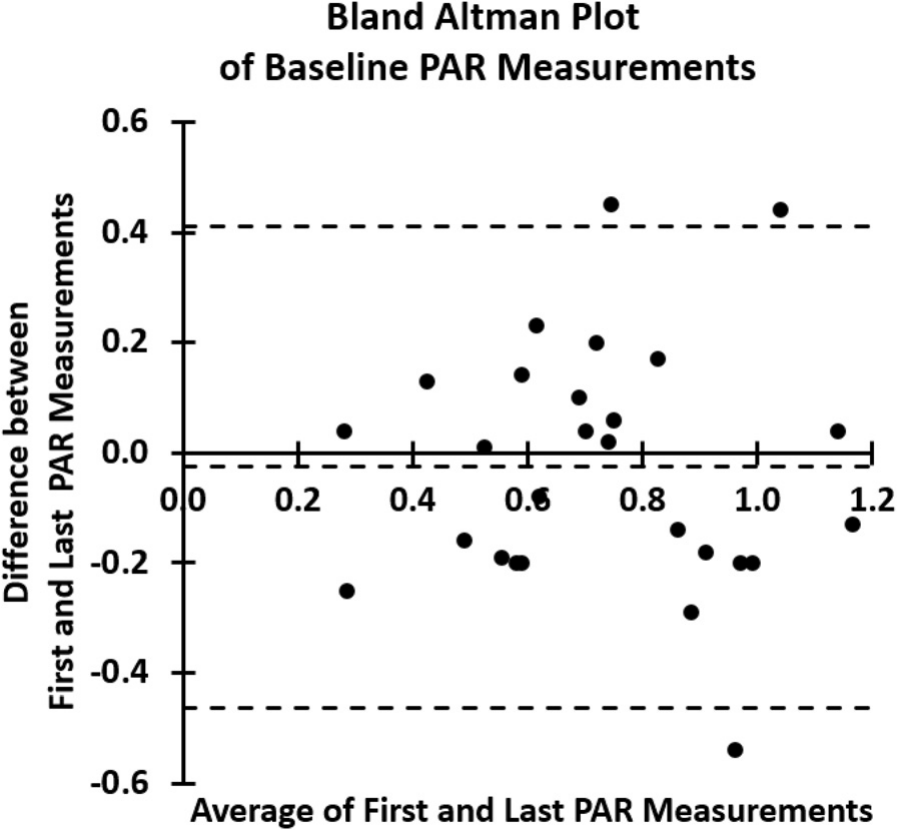
Bland-Altman plot of baseline Pulse Amplitude Ratio (PAR) measurements

Table 2 shows the changes in variables before and after hemodialysis. All continuous variables with normal distributions are presented as mean ± standard deviation. The average coefficient of variation was 0.14. Figure 2 shows a Bland-Altman plot of the first and last baseline PAR measurements in each subject.

**Table 2:** Characteristics before and after hemodialysis

	Before	After	P, After vs Before
	Hemodialysis	Hemodialysis	
Systolic Blood Pressure (mmHg)	138.7 ± 19.4	133.2 ± 25.0	*p* = 0.081
Diastolic Blood Pressure (mmHg)	79.8 ± 14.9	72.6 ± 13.6	*p* = 0.003*
Heart rate (beats per minute)	82.2 ± 12.8	80.4 ± 11.2	*p* = 0.221
Temperature (Fahrenheit)	98.0 ± 0.72	98.0 ± 0.81	*p* = 0.481
Weight (kg)	99.7 ± 36.9	97.0 ± 36.0	*p* < 0.0003*
Body Mass Index (kg / m^2^)	35.2 ± 13.7	34.3 ± 13.4	*p* < 0.0004*
Volume Removed (L)	------	3.07 ± 1.08	
Duration of Hemodialysis (minutes)	------	225.9 ± 31.6	
Range (min)		180–330	
Number of Successful Expiratory efforts	3.51 ± 0.71	3.82 ± 0.91	*p* = 0.152
Pulse Amplitude Ratio	0.74 ± 0.24	0.62 ± 0.23	*p* = 0.003*

Variables are presented as mean ± standard deviation. * *p* < 0.05 considered significant

**Fig. 3:**
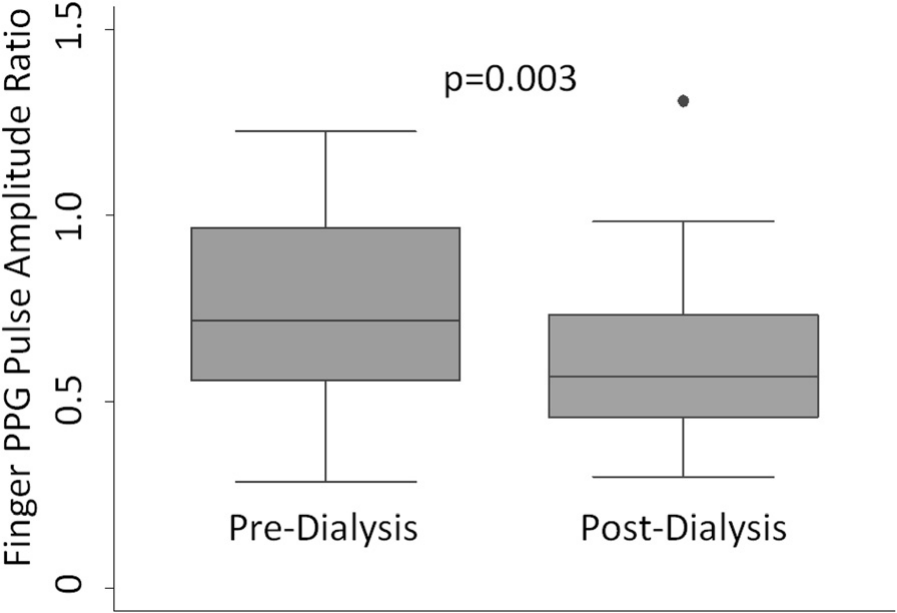
Box plots of Pulse Amplitude Ratio (PAR) by finger photoplethysmography (PPG), before and after hemodialysis. PAR decreased after one session of dialysis

There was a statistically significant change in diastolic blood pressure before and after hemodialysis (79.8 ± 14.9 mmHg to 72.6 ± 13.6 mmHg, *p* = 0.003); however, no other vital signs changes were statistically significant. Weight and body mass index changes did have significant changes as well (99.7 ± 36.9 kg to 97.0 ± 36.0, *p* < 0.0003; 35.2 ± 13.7 kg/m^2^ to 34.3 ± 13.4 kg/m^2^, *p* < 0.0004, respectively). The mean volume removed was 3.07 ± 1.08 L, with a range of 0.6 L to 5.0 L. Correspondingly, there was a significant decrease in PAR, from 0.74 ± 0.24 to 0.62 ± 0.23 (*p* = 0.003, Fig. 3). Furthermore, the change in PAR was correlated with baseline PAR (*r* = 0.48, *p* = 0.01). The change in PAR was not correlated with volume removed (*r* = 0.14, *p* = 0.33), volume removed per starting weight (*r* = 0.11, *p* = 0.60), change in weight (*r* = 0.17, *p* = 0.39), or relative change in weight (*r* = 0.12, *p* = 0.54). Also, the percent change in PAR was not correlated with volume removed (*r* = 0.07, *p* = 0.74), volume removed per starting weight (*r* = 0.12, *p* = 0.55), change in weight (*r* = 0.12, *p* = 0.53, or relative change in weight (*r* = 0.13, *p* = 0.51).

One patient with 3 years of hemodialysis requirement experienced significant muscle cramps, requiring hemodialysis to be discontinued 30 min before desired completion (targeted hemodialysis time 240 min; actual was 180 min). The patient’s weight decreased by 4.7 kg (179.6 kg to 174.9 kg) with a change in PAR 0.06. There was a fall in both systolic and diastolic blood pressures (from 137 to 108 mmHg systolic and from 75 to 55 mmHg diastolic). Heart rate did not change (93 beats per minute to 86 beats per minute). Of note the patient was on no anti-hypertensive medications.

## Discussion

In this study, we investigated whether a noninvasive index of left heart filling pressure using finger photoplethysmography during the Valsalva maneuver is sensitive enough to detect changes that occur after one session of hemodialysis. The index is the ratio of pulse amplitude at end-Valsalva to pulse amplitude at rest (Pulse Amplitude Ratio, PAR). We used a hand-held, battery-powered device with an automated graphical user interface that guides the subject and calculates PAR. We found that PAR measured by the device does change significantly with one session of hemodialysis. The implication is that the change in left ventricular filling pressure that occurs with volume removal during dialysis can be detected noninvasively. Therefore, it is worth investigating whether PAR can be used to detect residual left ventricular pressure overload between dialysis sessions, or whether PAR can predict intradialytic hypotension.

We also found that the change in PAR with hemodialysis correlated with baseline PAR; i.e., when pre-hemodialysis PAR was higher, the decrease in PAR with hemodialysis was greater. This suggests that when pre-hemodialysis LVEDP is higher, the decrease in LVEDP with hemodialysis is greater. This probably reflects the nonlinear pressure-volume relation in the left ventricle, where a given change in volume produces a greater change in pressure at higher starting points on the curve [18]. The correlation was not strong, probably in part because the pressure-volume curve is different for different subjects.

The change in PAR was not correlated with the amount of volume removed or with the change in weight. This is also probably due to the nonlinear pressure-volume relation. Each subject may have been on a different starting point on their pressure-volume curve, and each subject’s pressure-volume curve may differ from that of the others. Hence, a given volume removed with HD cannot predict the magnitude of the resulting decrease in LVEDP. This concept may help to explain some of the limitations of using volume and weight changes to manage dialysis patients.

Blood pressure responses to the Valsalva maneuver and their correlation with cardiac volume or pressure was first demonstrated several decades ago, and later by others [13–15, 19, 20]. The Valsalva maneuver increases intrathoracic pressure and with the transmission of the elevated intrathoracic pressure to the periphery, one sees a rise in blood pressure initially. If the maneuver is sustained for at least 10 s, the limitation on venous return will lead to a decrease in stroke volume, and thus, a decrease in peripheral pulse amplitude [15]. However, if the patient has elevated cardiac filling pressures due to volume overload (e.g. underdialyzed end-stage renal disease, congestive heart failure), then the sustained expiratory effort will not result in a decrease in stroke volume due to the reservoir of central blood volume [19, 20]. Therefore, in patients with end-stage renal disease on chronic hemodialysis, quantifying these changes may be useful in individualizing fluid removal goals.

PAR is a continuous variable, best measured using a continuous waveform. PAR has been shown to reflect left heart filling pressure using indwelling pressure transducers [15, 19] and noninvasive surrogates of a continuous blood pressure waveform [21–23]. We demonstrated the correlation between PAR and LVEDP using ordinary finger photoplethysmography and an expiratory effort of 20 mmHg, which is a milder effort than is used in other studies and can be achieved by more patients [17].

Other non-invasive techniques have been investigated for assessing changes after hemodialysis fluid removal. One technique is ultrasound Doppler imaging to evaluate the ratio of early transmitral flow velocity (E) to early diastolic mitral annular velocity (E’), because E/E’ has been shown to reflect left ventricular filling pressure [24, 25]. In a longitudinal study, E/E’, as well as serum proBNP, increased over time, suggesting that these indices might be useful in managing hemodialysis goals in order to limit chronic fluid overload [7]. However, echocardiography and serum variables would be impractical for assessing acute changes in hemodialysis patients. Other limitations of using echo Doppler indices are the cost of the machine and the need for a specialized ultrasonographer.

Lung ultrasound to detect pulmonary congestion (extravascular lung water) has been studied [9]. Lung water (and thus lung congestion) was reduced after dialysis but also could indicate residual congestion after dialysis. However, it is unclear what was involved in quantifying lung water. And, as with cardiac echocardiography, a highly trained operator is required.

Hand-carried ultrasound (HCU) to assess IVC diameter has been shown to predict patients who developed intradialytic hypotension [8]. HCU is much less expensive and more portable than standard echocardiography machines. Also, although training is required for proficiency in using HCU to assess IVC diameter, less training is required than to perform standard echocardiography. However, a photoplethysmography-based hand-held device would be much less expensive than HCU, and an automated patient user-interface and PAR calculation algorithm allow for minimal operator training.

Central venous oxygen saturation (ScO2) from dialysis catheters has shown promise in predicting intradialytic hypotension during hemodialysis [10]. There was a wider gap in ScO2 measurements between hypotension-prone and hypotension-resistant patients than the gap in blood volume measurements, the current standard tool for intradialytic monitoring. However, as the authors point out, the results are not generalizable to the dialysis population, most of whom have A-V fistulas.

Basso et al. compared several techniques for assessing the fluid status of chronic hemodialysis patients before and after hemodialysis, including bioimpedance spectroscopy, ultrasound lung comet score, B-type natriuretic peptide (BNP), and IVC diameter [26]. Each technique was able to detect changes in volume status with dialysis. Also, each detected hyperhydration before and after hemodialysis except for IVC diameter, and each detected hypovolemia before and after hemodialysis except for BNP. Interoperator reproducibility was high for each technique. Although BIA devices provide information about hydration status, extracellular water expansion as determined by BIA may not equate with plasma volume expansion [11].

The use of PAR based on a noninvasive waveform during the Valsalva maneuver was shown in one study to yield prognostic information in hemodialysis patients [4]. A noninvasive blood pressure waveform during the Valsalva maneuver identified future heart failure exacerbation. The investigators used a Finapres, a sophisticated and expensive device based on the volume clamp method, which employs photoplethysmography in a servo-controlled feedback of an inflatable finger cuff. Whether PAR using ordinary finger photoplethysmography has prognostic value in hemodialysis patients needs further investigation.

Our study had several limitations. First, the number of patients evaluated was relatively small, 27. Although a statistically significant change in PAR was demonstrated, a larger number of subjects will need to be studied to establish the utility of PAR by finger photoplethysmography in guiding volume management in hemodialysis patients. Second, we did not quantify the amount of urine the recruited patients typically make in a day. Third, although we have previously shown that PAR by finger photoplethysmography correlates with invasively measured LVEDP in patients undergoing a cardiac catheterization for clinical reasons [Silber], we did not invasively measure LVEDP in these dialysis patients. Since hemodialysis patients may have calcification of their vasculature, which may impact PAR values obtained on distal extremities, it would be useful in a future study to determine the PAR-LVEDP relation specifically in a cohort of dialysis patients undergoing clinical cardiac catheterization. Fourth, we did not investigate whether the type of hemodialysis access played a role in PAR values obtained (e.g. comparing AV-fistulas with catheters). Again, future studies with a larger population will be needed to explore this further.

## Conclusion

In conclusion, an index of left heart filling pressure (PAR) obtained noninvasively using finger photoplethysmography during the Valsalva maneuver is sensitive enough to detect reductions in filling pressure after fluid removal with hemodialysis. Further studies are warranted to determine the clinical utility of PAR to help guide individualized goal-setting of fluid removal in dialysis.
